# HMGA2 as a Critical Regulator in Cancer Development

**DOI:** 10.3390/genes12020269

**Published:** 2021-02-13

**Authors:** Behzad Mansoori, Ali Mohammadi, Henrik J. Ditzel, Pascal H. G. Duijf, Vahid Khaze, Morten F. Gjerstorff, Behzad Baradaran

**Affiliations:** 1 Immunology Research Center, Tabriz University of Medical Sciences, Tabriz 51656-65811, Iran; b.mansoori_lab@yahoo.com (B.M.); amohammadi@health.sdu.dk (A.M.); shahgoli@health.sdu.dk (V.K.); 2 Student Research Committee, Tabriz University of Medical Sciences, Tabriz 51656-65811, Iran; 3 Department of Cancer and Inflammation Research, Institute for Molecular Medicine, University of Southern Denmark, 5230 Odense, Denmark; hditzel@health.sdu.dk; 4 Aging Research Institute, Physical Medicine and Rehabilitation Research Center, Tabriz University of Medical Sciences, Tabriz 51656-65811, Iran; 5 Department of Oncology, Odense University Hospital, 5000 Odense, Denmark; 6 Academy of Geriatric Cancer Research (AgeCare), Odense University Hospital, 5000 Odense, Denmark; 7 Institute of Health and Biomedical Innovation, School of Biomedical Sciences, Faculty of Health, Queensland University of Technology, Brisbane, QLD 4102, Australia; p.duijf@uq.edu.au; 8 University of Queensland Diamantina Institute, The University of Queensland, Brisbane, QLD 4102, Australia

**Keywords:** HMGA2, cancer, cell cycle, apoptosis, DNA repair, MAPK, TGFβ, RKIP, miRNA

## Abstract

The high mobility group protein 2 (HMGA2) regulates gene expression by binding to AT-rich regions of DNA. Akin to other DNA architectural proteins, HMGA2 is highly expressed in embryonic stem cells during embryogenesis, while its expression is more limited at later stages of development and in adulthood. Importantly, HMGA2 is re-expressed in nearly all human malignancies, where it promotes tumorigenesis by multiple mechanisms. HMGA2 increases cancer cell proliferation by promoting cell cycle entry and inhibition of apoptosis. In addition, HMGA2 influences different DNA repair mechanisms and promotes epithelial-to-mesenchymal transition by activating signaling via the MAPK/ERK, TGFβ/Smad, PI3K/AKT/mTOR, NFkB, and STAT3 pathways. Moreover, HMGA2 supports a cancer stem cell phenotype and renders cancer cells resistant to chemotherapeutic agents. In this review, we discuss these oncogenic roles of HMGA2 in different types of cancers and propose that HMGA2 may be used for cancer diagnostic, prognostic, and therapeutic purposes.

## 1. Introduction

High mobility group (HMG) proteins are nonhistone, chromatin-associated molecules involved in maintenance and functional regulation of DNA, including the processes of replication, recombination, transcription, and DNA repair [1]. The proteins of this superfamily bend DNA upon binding to the minor groove and thereby contribute to transcriptional regulation and maintenance of the chromatin structure [2]. HMG proteins have extremely broad DNA sequence specificity, therefore supplying an exceptional mechanism for protein–DNA interactions [3]. The biological activities of HMG proteins are regulated through epigenetic mechanisms, which affect their interactions with DNA and other transcription factors. Consequently, this affects signaling pathways by recruitment of different transcription factors to the target gene promotors, which cause a specific phenotypic outcome.

**Figure 1:**
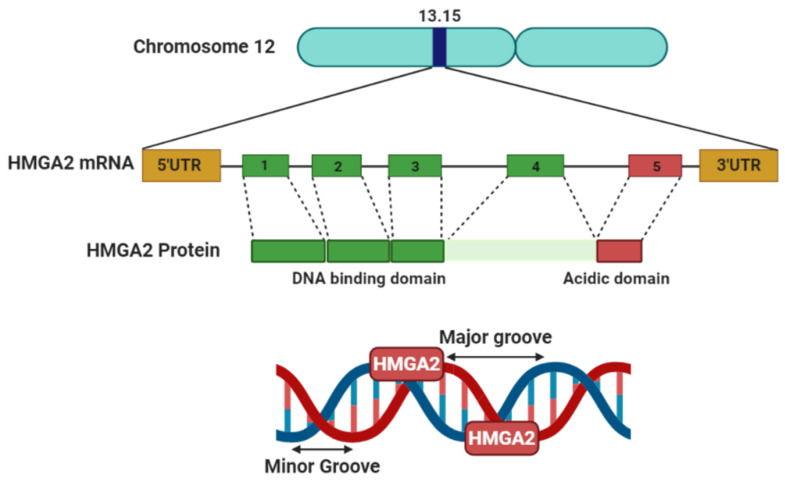
Schematic of the high mobility group protein 2 (HMGA2) gene and protein. Located on chromosome 12, the HMGA2 gene encodes a small protein, which can bind to AT-rich sites in the minor groove of DNA and subsequently regulate transcription by inducing conformational changes to the DNA.

The HMG superfamily is grouped into three main families, including HMGA, HMGB and HMGN. The HMGA gene family consists of two functional members, high mobility group A1 (HMGA1or HMGI/Y) and high mobility group A2 (HMGA2 or HMGI-C). HMGA proteins bind to AT-rich DNA regions that exist in short stretches in the minor groove of the DNA (Figure 1) [4]. In addition to DNA, they also interact with different proteins, such as E2 promoter-binding factor 1 (E2F1) and retinoblastoma protein (Rb). Increasing evidence indicates that HMGA proteins do not directly regulate transcriptional activity, but rather control gene expression by changing chromatin structure and organizing several transcription factors on a so-called “enhanceosome” [5,6]. When HMGA2 binds to AT-rich hooks of DNA, the conformation changes from disordered to ordered, and in turn this introduces bending, unwinding, straightening, and looping of DNA [7]. The acidic tail of the HMGA2 protein is responsible for protein–protein interactions. Thus, this domain mediates binding of HMGA2 to other DNA binding proteins, such as transcription factors [8].

Recent studies suggest a possible link between HMG proteins and cancer development [9,10]. HMGA2 is important during early embryogenesis and is highly expressed during oncogenesis. Studies have shown that HMGA2 increases cancer progression through the activation of several pathways. In this literature review, we highlight the oncogenic role of HMGA2 in tumor development and progression and discuss the probable signaling pathways affected by this architectural protein.

## 2. HMGA2 Structure and Function

The HMGA2 gene contains five exons distributed over a genomic area of more than 140 kb located at human chromosome 12q13-15 (Figure 1). The first three exons encode the AT-binding domain site, while exon 4 encodes a protein linker and exon 5 the acidic domain which does not activate transcription (Figure 1). HMGA2 is a rather small protein of less than 12 kDa, comprising 108 amino acids. Akin to the other protein family members, HMGA2 regulates gene expression by binding to the DNA minor groove and chromatin management [11].

The HMGA2 AT-binding domains are each comprised of nine amino acids, including the invariable repeat Arg-Gly-Arg-Pro [12]. During transcription, these domains can bind to B-shaped DNA and convert it from disordered to ordered conformations. Based on the number and location of the AT-rich binding sites, HMGA2 affects the conformation of DNA in several ways. By influencing the structure of DNA, it can change its effect on transcription by enhancing or suppressing the activities of numerous genes, consequently affecting various biological processes [6].

**Figure 2:**
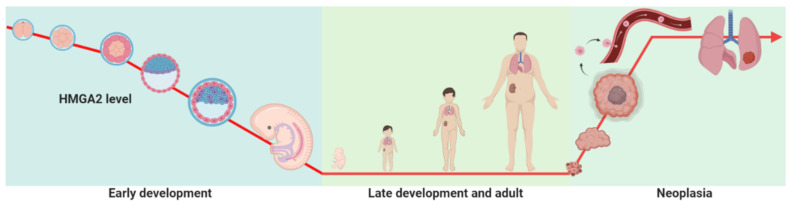
HMGA2 expression level during development and neoplasia. HMGA2 is highly expressed during early embryonic development. In contrast, in late development and during adulthood, the expression levels of this are low. However, the expression of HMGA2 is increased during the development of cancer.

HMGA2 is widely expressed in undifferentiated cells during early development and embryogenesis, but expression becomes more restricted as fetal development progresses. During late development and adulthood, it is expressed in kidney, liver, and uterus [13], while the expression is low in lung and kidney [14]. Importantly, HMGA2 is re-expressed in a variety of benign and malignant tumors including breast, lung, ovarian, colorectal, and pancreatic cancers [15,16,17] (Figure 2). A full genome analysis showed that HMGA2 is preferentially expressed by stem cells and that displayed a progressive decay in expression with age, partly due to increasing let-7b microRNA expression [18]. Furthermore HMGA2 expression has been limited to the mesenchyme during normal development [19]. The role of HMGA2 in adipogenesis, spermatogenesis, and stem cell development has been proved in in vivo experiments in an HMGA2 null mice model [20].

HMGA2 has been implicated in DNA translocations in lipomas and mesenchymal tumors. These generate chimeric transcripts in which exons 4 and 5 may be substituted by a wide range of other coding sequences such as *Snail1*, *Snail2*, and *Twist*, lead to binding of HMGA2 to a variety of genes with three AT-hooks [21]. These HMGA2 fusion proteins can both inhibit and activate gene expression by modifying chromatin arrangement, and some HMGA2 fusion proteins have been demonstrated to cause tumorigenicity. A truncated form of HMGA2 with only the three AT-binding domains and without an acidic domain was sufficient for transformation in vitro [22]. This finding supports other work showing that the acidic domain is not critical for triggering tumorigenicity [23]. Both full-length and 3′UTR truncated HMGA2 in transgenic mice develop benign mesenchymal tumors, including breast fibroadenomas and salivary gland adenomas [24]. Injection of HMGA2 overexpressing fibroblasts into athymic nude mice formed fibrosarcomas and metastases [25]. Furthermore, another study reported that both full-length and truncated HMGA2 induce the development of various benign tumors in mesenchymal tissues such as breast fibroadenoma, salivary gland adenoma, and preputial gland hyperplasia, suggesting that misexpression of HMGA2 itself is sufficient for tumorigenicity in this cell linage [24]. Recently, new insights into the role of HMGA2 translocations in tumorigenesis were provided. It was reported that a translocation leading to a reduction in the 3′UTR of HMGA2 might increase tumorigenicity [26]. HMGA2 has a long UTR (about 3000 nucleotides), which renders this mRNA amenable to regulation by multiple microRNAs (miRNAs). Reverse association of these miRNA and HMGA2 is crucial for regulatory mechanisms in both normal tissue development and tumorigenesis [26]. The hypothesis that miRNAs could regulate HMGA2 expression, as well as other oncogenes, was firstly confirmed by DICER knockdown in HeLa cells [26]. More recent studies identified various miRNAs which could regulate HMGA2 expression at the post-transcriptional level during oncogenesis by targeting the HMGA2 3′UTR. These include the *let-7 family* [27], *miR-330* [28], *miR-98-5p* [29], *miR-33-5p* [30], *miR-34a-5p* [31], *miR-497* [32], and *miR-491* [33].

## 3. HMGA2 Overexpression in Cancer

HMGA2 is aberrantly regulated in a broad range of human cancers, including lung [34], breast [35], and ovarian cancers [36] and increased expression of HMGA2 correlates with a higher risk of cancer progression. Comparing cancer to normal tissues by RNA-seq showed varying HMGA2 expression patterns in different types of cancers (Table 1). According to the datasets existing in the Oncomine cancer profiling database (www.oncomine.org, accessed on 7 Febuary 2021), HMGA2 is generally more highly expressed in sarcoma, brain and CNS, esophageal, head and neck, lung, melanoma, ovarian, and pancreatic cancers compared to corresponding normal tissue. In contrast, lower expression of HMGA2 was found in colorectal and gastric cancers, leukemia, and lymphoma (Table 1). In breast and prostate cancers, varying expression levels were observed in different studies (Table 1).

In addition to RNA-seq analyses, several studies that analyzed HMGA2 levels by IHC, Western blotting, and qRT-PCR found increased HMGA2 expression during cancer development. Previously, we demonstrated increased HMGA2 expression in breast tumor tissues compared to normal tissues [35,65]. Other studies showed that HMGA2 is overexpressed in lung [66,67], ovarian [36], breast [68], colorectal [69], pancreatic [70], gastric [71], prostate [72], bladder [73], tongue [74], pituitary [75], and thyroid [76] cancers. In contrast, one study reported that HMGA2 expression is reduced in nasopharyngeal cancer [77].

## 4. Regulation of HMGA2 by miRNAs in Cancer

**Figure 3:**
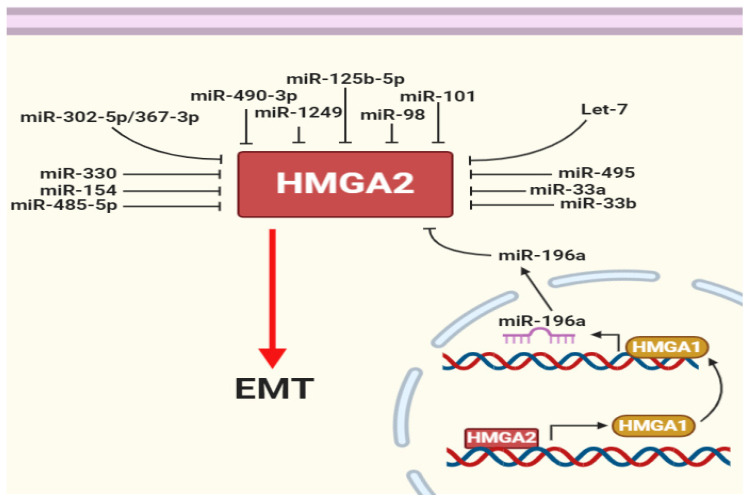
HMGA2 is regulated by multiple microRNAs. HMGA2 regulates itself by promoting HMGA1 and then HMGA1 decreased HMGA2 by increasing *miR-196a*. In addition, several microRNAs (miRNAs) have been shown to repress epithelial–mesenchymal transition (EMT) by targeting HMGA2.

miRNAs are master gene expression regulators at the post-transcriptional level. These small regulatory molecules bind to the 3′UTRs of specific target mRNAs. An endonuclease complex accumulates at the bound region. Consequently, the complex cleaves a target mRNA from the binding region or suppresses mRNA translation by trapping it in processing bodies (p-bodies) [78,79]. HMGA2 has a long 3′UTR, therefore providing a long targeting region for different miRNAs. Indeed, interactions of miRNAs with specific target mRNA is predicted using different databases, including miRanda, Pictar, miRTarbase and TargetScan. *Let-7* downregulation and HMGA2 overexpression occur during lung cancer development. The *let-7* family of miRNAs were the first reported miRNAs targeting the 3′UTR of HMGA2 and degrading HMGA2 mRNA [26]. Various databases predict eight putative target sites for *let-7*. Stimulation of HMGA2 influences the other family member, HMGA1. This induces *miR-196a* via a negative feedback loop that suppresses HMGA2 [80,81] (Figure 3).

In our recent study, we showed that *miR-330* targets HMGA2 and stable induction of *miR-330* expression inhibits Snail1 and increases E-cadherin expression in breast cancer cells [28]. Other studies have demonstrated that a range of other miRNAs, including *miR-101* [82], *miR-98* [83], *miR-302a-5p/367-3p* [84], *miR-1249* [85], *miR-490-3p* [86], *miR-125b-5p* [87], *miR-495* [87], *miR-485-5p* [88], *miR-33a* [89], *miR-33b* [90], and *miR-154* [72], target HMGA2 and reduce epithelial–mesenchymal transition (EMT) in cells in which these miRNAs are induced (Figure 3). In mouse xenografts, it was further shown that HMGA2 promotes breast tumor growth, survival, and metastasis by inducing *syndecan-2* (SDC2) via a *miR-200*-independent mechanism [91].

## 5. HMGA2 Induce Cancer Proliferation

HMGA2 influences the expressions of a broad spectrum of genes, many of which are involved in cancer cell proliferation and survival [92]. In this section, we discuss the mechanisms of HMGA2 in regulating cell proliferation with insights into the role of HMGA2 in the cell cycle, apoptosis and DNA damage repair.

### 5.1. HMGA2 Increase Cancer Cell Proliferation by Directing Cell Cycle

Uncontrolled cell cycle progression promotes neoplastic transformation. It was shown that increased HMGA2 expression in cancer supports cell proliferation by accelerating cell cycle progression. In accordance with this, HMGA2 silencing leads to cell cycle arrest in different phases of the cell cycle. For example, HMGA2 knockdown arrests cells in the G1 phase in ovarian cancer [36], but in the G2/M phase in leukemia [93]. In earlier studies, we demonstrated G2/M arrest in both breast and colorectal cancer in response to HMGA2 knockdown [35,94]. The direct binding of HMGA2 to the cyclic adenosine monophosphate (cAMP)-responsive component of cyclin A2 dislocates p120E4F complexes from cyclic AMP-responsive element (CRE), and accelerates the binding of the ATF/CREB complex to enhance cell cycle progression [95,96]. Furthermore, the activator protein-1 (AP1) complex, composed of Jun (JUN, JUNB, and JUND), FOS (FOS, FOSB, and FRA1) and FRA2 proteins, is crucial for the regulation of cell proliferation [97] and downregulation of HMGA2 inhibits the expression of JUNB and FRA1 completely [98]. In contrast, increased expression of HMGA2 enhances expression of these genes [98]. Moreover, HMGA2-mediated promotion of AP1 increases the expression of cyclin A2 to support cell cycle progression [98].

**Figure 4:**
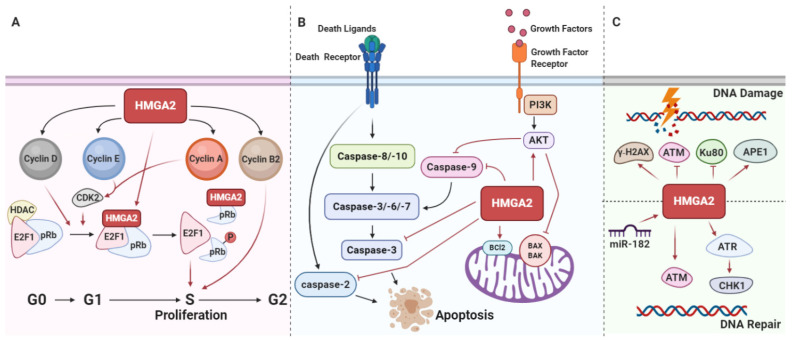
HMGA2 promotes cancer cell proliferation, inhibits apoptosis, and differentially affects DNA damage repair. (**A**) HMGA2 activates cyclin D1, cyclin E, and cyclin A and promotes pRB phosphorylation. This releases E2 promoter-binding factor 1 (E2F1), enabling it to induce transcription of target genes that promote S phase entry. HMGA2 directly activates E2F1 by replacing histone deacetylase 1 (HDAC1) from pRB. HMGA2 also triggers proliferation by inducing cyclin B2. (**B**) HMGA2 negatively regulates proapoptotic proteins, including caspase-2, -3, and -9, and induces antiapoptotic Bcl2. HMGA2 also promotes AKT signaling, which suppresses BAX/BAK and caspase-9 activation. (**C**) HMGA2 triggers γ-H2AX and human AP endonuclease 1 (APE1) and inhibits Ku-80 and ataxia telangiectasia mutated protein (ATM), thereby promoting DNA damage. In contrast, by activating ATM, ataxia telangiectasia, and Rad3-related kinase (ATR), and checkpoint kinase 1 (CHK1), HMGA2 also promotes DNA repair. *miR-182* is an oncogenic miRNA that promotes DNA repair by inducing HMGA2.

By interactions with E2F1 transcription factors, the pRB precisely regulates cell cycle entry into the S phase [99] (Figure 4A). Before cells enter the S phase, pRB phosphorylation and its inactivation cause E2F1 release, resulting in S phase entry [100]. It has further been demonstrated that HMGA2 supports the role of E2F1 as a transcription factor by dislocating histone deacetylase 1 (HDAC1) from E2F1 target promoters [75] (Figure 4A). The two most important cyclin-dependent kinase inhibitors (CDKIs) which restrict release of E2F1 from pRB are P16INK4A and p21CIP1/WAF1. Interestingly, HMGA2 overexpression directly activates the PI3K/AKT/mTOR/p70S6K signaling pathway, resulting in cyclin E activation and inhibition of p16INK4A and p21CIP1/WAF1 activities [101]. Additionally, RB phosphorylation is accelerated by the activation of cyclin D1/CDK4/CDK6 and HMGA2 supports the cyclin D1 synthesis [102]. This suggests that HMGA2 also influences cell cycle progression via the cyclin D1/CDK4/CDK6/pRB-E2F1 axis [103,104] (Figure 4A). In addition, HMGA2 could regulate cyclin B2 expression by binding to the promoter of the *CCNB2* gene and increasing its expression, thereby promoting G2/M transition and cell proliferation [105,106].

### 5.2. HMGA2 Protects Cancer Cells from Apoptosis

There is considerable evidence that HMGA2 can inhibit apoptosis in tumor cells and promote tumor growth. Tumors with high HMGA2 expression exhibit high proliferation and low apoptosis rates compared to tumors with low HMGA2 expression [65,107,108] and a mechanism for HMGA2 regulation of apoptosis has been proposed. It was shown that HMGA2 silencing promotes apoptosis by reducing antiapoptotic Bcl-2 expression in ovarian cancer [34] and we found that HMGA2 derepresses the expression of Bcl-2 by inhibiting *miR-34a* in breast cancer, thereby promoting the Bcl-2 antiapoptotic pathway [65]. Furthermore, there is positive feedback between the expression of Bcl-2 and HMGA2, as overexpression of Bcl-2 increases the expression of HMGA2 in thyroid cells [107]. The PI3K/AKT signaling pathway is often hyperactivated in human cancer and this contributes to resistance to apoptosis [108]. Activated AKT inhibits apoptosis by reducing caspase-9 and BAD activation [109,110]. Importantly, HMGA2 suppresses apoptosis by activating PI3K/AKT signaling, thereby reducing caspase-9 and BAD activation [111] (Figure 4B). Additionally, phosphorylation of ataxia telangiectasia and Rad3-related kinase (ATR)/CHK1 is considerably decreased during apoptosis. It has been shown that repression of HMGA2 inhibits the ATR/checkpoint kinase 1 (CHK1) signaling pathway, resulting in apoptosis [112]. In addition, silencing of TNF-related apoptosis-inducing ligand-R2 (TRAIL-R1) considerably enhances the level of let-7 and this decreases HMGA2 expression [113].

Intriguingly, apoptotic effects of HMGA2 have also been described. Caspase-2 promotes the release of cytochrome from mitochondria, which is essential for the induction of apoptosis [114]. Therefore, it is of interest that induction of HMGA2 expression upregulates levels of cleaved caspase-2, thereby inducing apoptosis [115] (Figure 4B). In another study, HMGA2 was implicated in inducing apoptosis by triggering 6-O-methylguanine-associated DNA damage [116]. Altogether, the above findings specify both anti- and proapoptotic roles of HMGA2.

### 5.3. HMGA2 Is Involved in DNA Damage and Repair Responses

HMGA2 influences the DNA repair process by controlling multiple DNA repair-associated proteins. It was demonstrated that HMGA2 possesses intrinsic apurinic/apyrimidinic (AP) site cleavage activity [117]. A physical interaction between human AP endonuclease 1 (APE1) and HMGA2 in cancer cells was demonstrated, which supports the involvement of HMGA2 in the base excision repair (BER) machinery [117]. HMGA2 has a positive and/or negative effect on nonhomologous end-joining (NHEJ). HMGA2 suppresses NHEJ and impairs DNA-PK dynamics by altering Ku70 and Ku80 binding to DNA ends [118]. HMGA2 caused the persistence of γ-H2AX, which may disrupt NHEJ. On the other hand, HMGA2 enhances NHEJ by activating the ataxia telangiectasia mutated protein (ATM) [119]. HMGA2 acts as a substrate for ATM and its downstream tumor suppressor checkpoint kinase 2 (CHK2), which are crucial for DNA damage signaling. Exposure to genotoxic radiation or chemicals, such as UV irradiation and peroxide (H_2_O_2_), increases the expression of HMGA2, which correlates with enhanced ATM expression and an increased DNA damage response [120]. In addition, interaction between HMGA2 and the ataxia telangiectasia and Rad3-related kinase (ATR) promotes ATR-checkpoint kinase 1 (CHK1) signaling pathway activation, which in turn induces G2/M cell cycle arrest. This promotes chemoresistance against alkylation-induced genotoxicity in different types of human cancer [121]. In addition, there is some evidence to show that, during DNA damage, HMGA2 expression induces and subsequently sustains ATR and CHK1 levels, which prolongs G2/M arrest and enhances tumor cell survival in different human tumors with high HMGA2 expression [121] (Figure 4C).

It has been shown that protein phosphatase 4 regulatory subunit 1 (PP4R1) regulates centrosome maturation, DNA repair, apoptosis, and tumor necrosis factor (TNF) signaling. It was reported that PP4R1 could cooperate with HMGA2 and promote EMT through activating MAPK/ERK signaling pathway in lung cancer cells [122]. HMGA2 could promote nucleotide excision repair by binding to the AT-rich site located 298-323 upstream of the excision repair cross-complementation group 1 (ERCC1) gene transcription start site [123]. Oncogenic *miR182* impairs DNA double-strand breaks repair; it may do this through downregulation of *BRCA1* and *MTSS1* and upregulation of HMGA2 and *γH2AX* expression (Figure 4C) [124].

### 5.4. HMGA2 Is Involved in Phenotype of Cancer Stem Cells

Cancer stem cells (CSCs) are a minor population of cells that exist in tumor tissues. They have the abilities of self-renewal, differentiation, tumor initiation, and drug resistance [125]. These cells are assumed to persist in tumor tissues as a distinct population that may trigger relapse. HMGA2 has been shown to be involved in cancer stemness in different types of cancers [126,127,128,129]. Our recent study demonstrated that HMGA2 and CD133 positively correlate in breast tumors. In this study, we reported that HMGA2 knockdown decreases the number of cancer colonies, as well as reduces the size of mammospheres. We also showed that HMGA2 suppression reduces the population of CD133+ and CD44+ cells [35]. HMGA2 suppression decreases expression of the cancer stem cell markers ALDH, SOX2, and Nanog [130]. Furthermore, HMGA2 expression increases more than 9-fold in CD133-positive population of glioblastoma multiforme (GBM) neurosphere cells compared to CD133-negative cells. In contrast, HMGA2 knockdown reduces GBM stemness [131]. HMGA2 is a specific modulator of neural and embryonic stem cells’ (ESCs) self-renewal potentials [31]. HMGA2 directly binds to the *SOX2* promotor and regulates the expression of this gene, which encodes an important cancer stem cell marker [132]. In addition, another study demonstrated the positive regulatory role of HMGA2 on the SOX2 function in anaplastic astrocytoma side population cells [129]. A recent study indicated that the lin-28B-let-7-HMGA2 signaling axis controls the high self-renewal potential of fetal hematopoietic stem cells [18]. Additionally, HMGA2 increases the expression of cancer stem cell markers, including CD44, Oct4, and Twist1 [133]. There is a positive correlation between HMGA2 and CD44 in gastric tumors. HMGA2 overexpression increases the gastric cancer spherocytes, as well as expression of the markers CD44, ALDH1, SOX2, and Oct4 [134]. In contrast, HMGA2 seems to be a negative regulator of both Ink4a and Arf expression. HMGA2 binds to the JunB locus and subsequently promotes Ink4a/Arf expression and self-renewal of stem cells. Restoration of low let-7 expression results in the HMGA2 downregulation and increases Ink4a/Arf, triggering p16INK4a expression in self-renewing cells. Deletion of HMGA2 in mice reduces stem cell numbers and self-renewal. Moreover, p16(Ink4a) and p19(Arf) expression are increased in HMGA2-deficient stem cells [31,135]. HMGA2 promotes self-renewal of neural stem cells (NSCs) by negative regulation of Ink4a/Arf expression [31]. It also regulates expression of FOXM1 and PLAU, which improves self-renewal and invasiveness of glioma-initiating cells [136]. Yamazaki et al. showed a positive correlation between HMGA2 and BMI-1-1 in head and neck squamous cell carcinoma (HNSCC). BMI-1-1 and HMGA2 promote self-renewal in stem cells via negative regulation of the expression of the tumor suppressors Ink4a and Arf [137]. The proinflammatory signals in M1 macrophages induce stemness properties in nonstem breast cancer cells through STAT3/NF-κB signaling via activation of lin-28B/let-7/HMGA2. It was suggested that suppression of HMGA2 expression directly overturns the proinflammatory signals, inducing the expression of the reprogramming factors Klf-4 and Nanog, repressing mammosphere formation, and reducing the ALDH1+ subpopulation [85]. HMGA2 expression is increased in chemotherapy-naive samples. However, there are no HMGA2 expression differences between chemotherapy-naive and postchemotherapy samples [138]. Finally, in gastric cancers, by targeting both *Bcl2* and HMGA2, *miR-34a* suppresses self-renewal and differentiation [139].

## 6. HMGA2 Promotes Angiogenesis during Cancer Development

Angiogenesis is the formation of new capillaries from pre-existing blood vasculature. It is a crucial mechanism required for a number of pathophysiological events. During tumor growth, angiogenesis is required for delivery of oxygen and nutrients and removal of metabolic waste products from tumor sites [140]. There are data suggesting that HMGA2 may regulate angiogenesis in tumor formation.

It was demonstrated that silencing of HMGA2 reduced the angiogenesis in mouse endothelial progenitor cells, and, conversely, HMGA2 induction enhanced angiogenic functions [141]. In another study, HMGA2 was showed to promote angiogenesis in oral squamous cell carcinoma. The results from HMGA2-bound DNA fragments enriched by ChIP assay confirmed the involvement of HMGA2 in VEGF signaling and TGF-b signaling pathways [142]. In addition, Li et al. showed that HMGA2 increases angiogenesis in leiomyoma cells and myometrial (MM) xenografts via inducing angiogenic factors including VEGFA, EGF, bFGF, TGFa, VEGFR1, and VEGFR2, augments HUVEC tube development, and increases IGF2BP2 and pAKT levels [143]. Xia et al. showed HMGA2, in cooperation with nuclear factor-kB (NF-kB), binds to the AT-rich regulatory region of the IGF2BP2 gene [144].

It was shown that metformin decreases angiogenesis and tumor growth by reducing HMGA2 levels in cervical cancer cells through regulation of *miR-142-3p*. Metformin was suggested to prevent *miR-142* sponging to allow targeting of HMGA2 [145]. It was further reported that *miR-1249* suppresses colorectal cancer (CRC) angiogenesis by targeting both VEGFA and HMGA2 through the AKT/mTOR signaling pathway [146]. In another study, let-7 was shown to inhibit angiogenesis by targeting IL-6, HMGA2, and VEGF expression [147]. Thus, together, these results suggest a role for HMGA2 in angiogenesis.

## 7. HMGA2 Increases EMT, Invasion, and Metastasis

During the epithelial–mesenchymal transition (EMT) process, epithelial-like cells lose their polarities and cell–cell adhesion and transdifferentiate into mesenchymal-like cells, thereby gaining invasive and migratory properties. EMT enables cancer cells to metastasize to other organs via blood or lymphatic vessels [148]. EMT starts with alterations of the expression of genes that repress epithelial properties and induces mesenchymal features, including reduced E-cadherin levels and increases levels of vimentin, Snail1/2, ZEB1/2, and Twist. It has been shown that HMGA2 induces expression of mesenchymal markers and decreases the expression of epithelial markers, thereby increasing invasion and metastasis [35,85]. Furthermore, suppression of HMGA2 in vitro significantly inhibits cancer cell mobility and the expression of EMT hallmark proteins. Below, we discuss the role of HMGA2 in several pathways involved in EMT.

### 7.1. The MAPK/ERK Pathway

**Figure 5:**
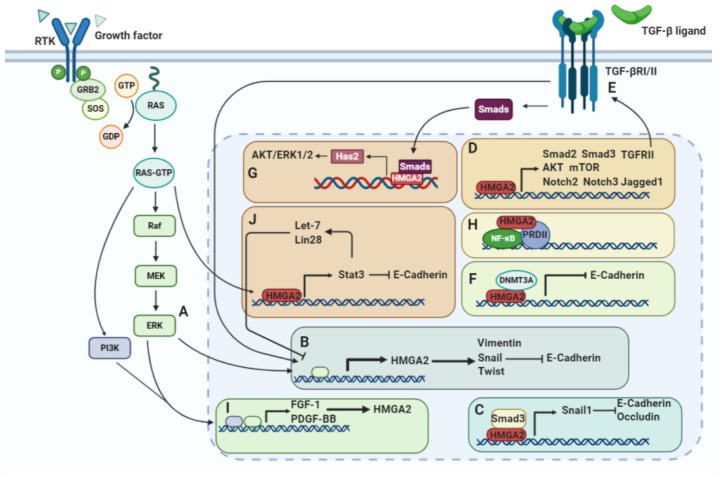
HMGA2 induces EMT by activation of different signaling transducers. (**A**) HMGA2 expression is induced by the RAF/MEK/ERK pathway. (**B**) HMGA2 promotes the expression of vimentin, Snail and Twist and reduces E-cadherin expression. (**C**) Colocalization of HMGA2 and smad3 on the Snail1 promotor induces expression of Snail1, and then Snail1 inhibits E-cadherin and occludin expression. (**D**) HMGA2 controls activation of the TGFβ, MAPK, and Notch pathways by regulating the main elements, including Smad2, Smad3, TGFβRII, AKT, mTOR, Notch2, Notch3, and Jagged1. (**E**) TGFβRII induced by HMGA2 increases activity of the TGFβ signaling pathway, which promotes HMGA2 expression in a positive feedback loop. (**F**) Long-term TGFβ signaling activation causes HMGA2 to recruit DNMT3A to the E-cadherin gene promoter and inactivates its transcription by DNA methylation. (**G**) During TGFβ pathway signaling, Smads cooperate with HMGA2 to bind to the Has2 promoter, inducing expression of Has2, which then activates AKT/ERK1/2 signaling. (**H**) HMGA2 enhances the binding of NFκB to the positive regulatory domain II (PRDII) transcription factor. (**I**) Regulators downstream of the MAPK and PI3K pathways, including FGF-1 and platelet-derived growth factor-BB (PDGF-BB), induce HMGA2 expression. (**J**) HMGA2 induces the expression of STAT3. Then, STAT3 activation promotes EMT by reducing E-cadherin expression. STAT3 also activates the miRNAs *let-7* and *lin-28*, which suppress HMGA2 expression.

The RAF/MEK/ERK pathway, also known as the MAPK pathway, induces HMGA2 expression (Figure 5A). HMGA2 overexpression was found to increase the level of phosphorylated ERK. Additionally, inhibiting the MAPK signaling pathway using the MAPK inhibitor U0126 decreases HMGA2 expression and antagonizes HMGA2-mediated EMT and migration [149]. Watanabe et al. demonstrated that HMGA2 silencing increases E-cadherin and decreases Vimentin levels. Snail is a transcriptional repressor of E-cadherin whose levels decreased after HMGA2 silencing in pancreatic cancer cells (Figure 5B). The authors also suggested that HMGA2 activates the Snail gene promoter by binding to the upstream AT-rich region [70]. In another study, Hawsawi et al. showed that HMGA2 is overexpressed in aggressive prostate cancer cell lines and demonstrated that HMGA2 increases the levels of mesenchymal factors, including Snail, Twist, and vimentin, while decreasing epithelial factors. They further showed that HMGA2 overexpression increases E-cadherin translocation from the cell membrane into the cytoplasm and/or nucleus. Thereby, the cells becoming invasive or metastatic [149,150]. HMGA2 promotes metastasis by induction of osteopontin and CXCR4 [91].

**Figure 6:**
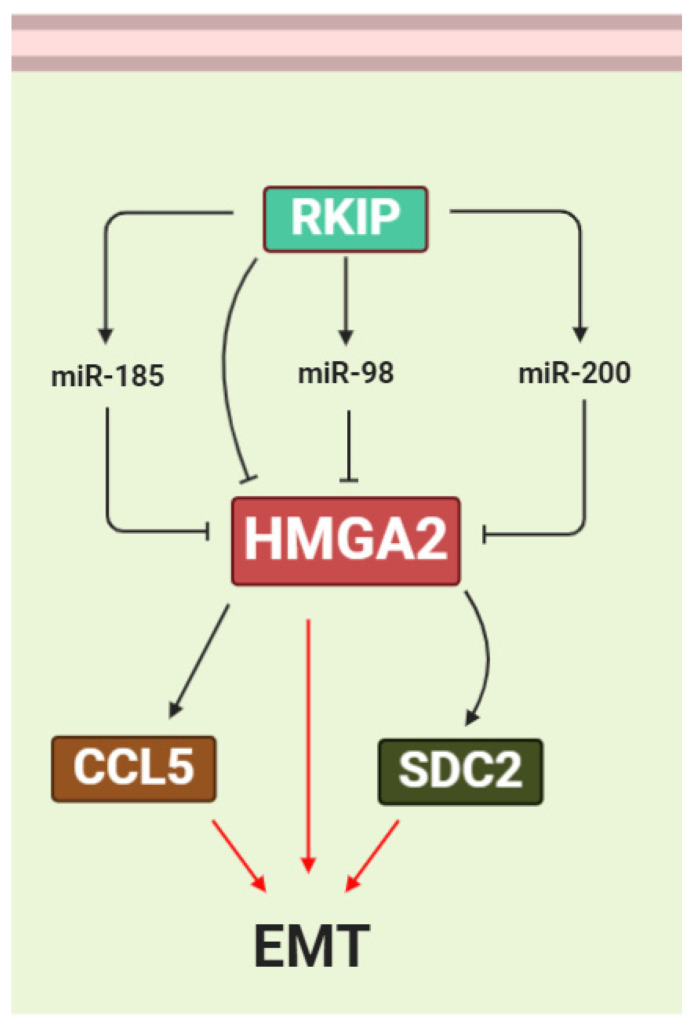
HMGA2 is regulated by the RAF kinase inhibitor protein (RKIP) pathway. RKIP suppresses HMGA2 expression directly, as well as indirectly, by inducing the miRNAs *miR-185*, *miR-200*, and *miR-98*, which target the 3′UTR of HMGA2 mRNA. Suppression of HMGA2 via RKIP and miRNAs reduces the expression of the EMT-promoting factors CCL5 and SDC2.

The RAF/MEK/ERK signaling cascade induces HMGA2 expression. Activation of RAF-1 increases HMGA2 expression in Pa-4 ovarian cancer cells [151]. The RAF kinase inhibitor protein (RKIP) suppresses the expression of many prometastatic genes in triple-negative breast cancer (TNBC) cells by reversing the biological activities of HMGA2 [152,153]. RKIP has been shown to suppress the expression of many prometastatic genes in TNBC cells by inhibiting HMGA2 expression in the mammary tumors. It was suggested that RKIP suppresses cell C-C motif chemokine ligand 5 (CCL5) expression, macrophage recruitment, and metastasis via coordinated HMGA2 signaling [154] (Figure 6). The HMGA2/*miR-200b*/LOX axis plays an important role in the initial stages of breast tumor cell invasion and metastasis. *MiR-200* is induced by *RKIP* and downregulates HMGA2 expression. *MiR-200* directly inhibits *Lysyl oxidase* (LOX) expression, leading to inhibition of breast tumor invasion [91] (Figure 6). Zou et al. suggested that *miR-185* targets HMGA2 via the RKIP pathway and inhibits breast cancer cell growth and invasion. They showed that overexpression of *RKIP* upregulates *miR-185* expression and that HMGA2 is one of the targets for this miRNA [155] (Figure 6). In another study, Chen et al. reported that overexpression of *RKIP* upregulates *miR-98* expression and this inhibits glioma cell invasion via targeting of HMGA2 [156] (Figure 6).

### 7.2. TGFβ/Smad Signaling

Similar to Snail1 and ZEB1, TGF-β also transcriptionally induces HMGA2 expression [157] (Figure 5B). Thuault et al. demonstrated that HMGA2 is induced by the TGF-β pathway during the EMT process. They also showed that Smad signaling induces HMGA2 expression, responsible for the aggressiveness of cancer cells; in addition, they reported HMGA2 may regulate expression of Snail, Slug, δEF-1/ZEB-1, and SIP-1/ZEB-2 as well as Twist and E-cadherin during EMT [91]. Our recent study demonstrated that HMGA2 suppression inhibits breast cancer EMT by affecting the TGF-β pathway and reducing the Smad3 transducer to further reduce Snail1 levels [35]. HMGA2 directly binds to the Snail1 promotor or colocalizes with smad3 on the Snail1 promotor. In addition, HMGA2 increases the physical interaction between Smad3 and the Snail1 promotor [158] (Figure 5C). Subsequently, Snail1 not only reduces expression of the epithelial-related genes, including E-cadherin, occluding, and coxsackie adenovirus receptors, but also increases expression of mesenchymal-related genes [159]. HMGA2 expression is upregulated by TGF-β activation, as shown by lentiviral infection and pharmacological assays. Furthermore, HMGA2 binds to an AT-rich area on the Twist1 promotor and enhances its transcription. Concomitant silencing of Twist1 and Snail1 revert mesenchymal HMGA2-expressing cells to a more epithelial phenotype compared to silencing Snail1 or Twist1 individually [160]. TGF-β signaling, via induction of Smads, increases HMGA2 expression. Subsequently, HMGA2 increases Snail and Twist expression by binding to their promotors. Finally, the overexpression of Snail1 and Twist1 increases ZEB1, ZEB2, and Snail2, which causes EMT reprograming. In another study, it was shown that HMGA2 induces Snail2 expression by directly binding to the Snail2 promoter. In addition, silencing of Snail2 reverses HMGA2-induced EMT and decreases invasion and migration of colon cancer cells [161]. Hou et al. reported that blocking of EMT using a selective inhibitor of TGFβ/Smad, TGFβ/ERK or Notch signaling pathway significantly inhibits HMGA2 protein expression. In addition, HMGA2 silencing impairs TGFβ2-associated phosphorylation of Smad2 and Smad3. In addition, HMGA2 suppression inhibits the upregulation of Jagged1, Notch2, and Notch3 induced by TGFβ2 [162] (Figure 5D). Long-term TGFβ signaling activation causes HMGA2 to recruit DNA methyltransferase (DNMT3A) to the E-cadherin gene and silencing of this gene by DNA methylation, thereby increasing the EMT phenotype [163] (Figure 5F).

Kolliopoulos et al. showed that TGFβ binding to its receptors (TβRI and TβRII) initiates signaling via Smad-dependent and -independent signaling pathways. Smads cooperate with HMGA2, resulting in Has2 transcription and production of hyaluronan. In turn, hyaluronan binds to CD44, triggering the AKT and ERK1/2 signaling pathways, consequently inducing EMT and breast cancer progression [164] (Figure 5G). Moreover, TGFβ beta receptor 2 (TGFβRII), induced by HMGA2, promotes an autocrine TGFβ signaling feed-forward positive loop in EMT. As HMGA2 increases TGFβRII, it activates TGFβ signaling, increasing HMGA2 expression levels, following which HMGA2 itself promotes TGF signaling [165] (Figure 5E).

### 7.3. The PI3K/AKT/mTOR Pathway

Activation of the PI3K/AKT/mTOR signaling pathway is crucial for EMT processes, which includes cancer cells invasion and migration [166]. Tan et al. reported that HMGA2 silencing decreases proliferation of acute myeloid leukemia cells by reducing phosphorylation of AKT and mTOR (Figure 5D) [93]. The fibroblast growth factor-1 (FGF-1) and platelet-derived growth factor-BB (PDGF-BB) receptors are known to signal through both the MAPK pathway and the PI3K pathway. Ayoubi et al. showed that FGF-1 and PDGF-BB can induce HMGA2 expression [167] (Figure 5I). In another study, they showed that treatment of 3T3-L1 cells with the inhibitor of PDGF alpha and beta receptors, Tyrphostin AG1296, results in a strong inhibition of HMGA2 mRNA expression by serum. In addition, treatment of cells with the EGF receptor tyrosine kinase inhibitor Tyrphostin AG1478 leads to a slight inhibition of HMGA2 mRNA expression. The PI3-kinase inhibitors wortmannin and LY294002 significantly inhibit the induction of HMGA2 mRNA by serum [168]. Thus, these studies provide strong evidence for the involvement of this pathway in the regulation of HMGA2 expression.

### 7.4. The NFκB Pathway

The first cooperation between the HMGA family and NFκB was reported by Thanos et al. They showed that HMGA1, in conjunction with NFκB, induces IFN-β expression in virus infection immunity [169]. Noro et al. showed that HMGA2 physically interacts with the p50/p65 subunits of NF-κB [170]. HMGA2 is able to enhance the binding of NFκB to the positive regulatory domain II (PRDII) transcription factor (Figure 5H) [171]. It was shown that HMGA2-dependent expression of IMP2 plays an important role during embryonic development and in tumorigenesis. The authors showed that the mRNA expression level of IMP2 increased nearly two-fold in HMGA2-induced cells compared to control cells [172]. Additionally, they showed that the AT-rich regulatory region resides in the first intron of the insulin-like growth factor 2 mRNA binding protein 2 (IGF2BP2/IMP2) gene. The AT-rich regulatory region mimics the response of endogenous *IMP2* to HMGA2. This promotes HMGA2 binding both in vitro and in vivo. A consensus NF-κB binding site was identified immediately adjacent to the AT-rich regulatory region that binds to NF-κB. Via this mechanism, NF-κB and HMGA2 cooperate to regulate IMP2 gene expression [6,173].

### 7.5. STAT3 Signaling

Inflammatory cytokines can stimulate EMT by activation of STAT3. STAT3 acts as a transcription factor for Twist, Snail, and ZEB1, increasing their expression by binding to their promoters [174,175]. Additionally, ectopic expression of STAT3 suppresses E-cadherin expression in prostate cancer [176]. It was reported that HMGA2 activates STAT3 through the Ras signaling pathway. This is necessary for EMT in salivary epithelial cells [177]. In addition, the miRNAs lin-28 and let-7 have been shown to regulate the STAT3 signaling pathway. Moreover, HMGA2 is one of the targets for let-7. The existence of a dynamic switch mechanism was proposed. This involves a lin-28/let-7/HMGA2 signaling circuit that controls the STAT3-mediated inflammatory response, which may regulate self-renewal and differentiation in cancer stem cells [178] (Figure 5J).

### 7.6. HMGA2 Is Involved in Other Signaling Pathways

It was demonstrated that expression of the oncogenic HMGA2 leads to Ten-eleven translocation methylcytosine dioxygenase 1 (TET1) suppression. TET1 was described as binding and demethylating itself as well as HOXA7 and HOXA9, and reduced TET1 expression level causes TET1 inhibition along with loss of HOXA7 and HOXA9 expression. Suppression of TET1 and HOXA9 promotes breast cancer development and metastasis by increasing oncogenes [179].

One of the key pathways which involves in the initiation and progression of most types of cancers is Hippo-YAP signaling [180]. It was observed that interfering HMGA2 cased YAP protein reduction. Hence, they showed that the effect of HMGA2 on TNBC is related to targeting of YAP. These results showed that HMGA2 could stabilize the YAP protein via impeding its ubiquitination in the proteasome system [181].

## 8. HMGA2 Increase Cancer Drug Resistance

The ability of cancer cells to survive and proliferate even in the presence of anticancer therapeutic agents is called cancer drug resistance. The presence of cancer stem cell populations in tumor tissues promotes the probability of chemotherapy resistance. There are two possible scenarios of cancer drug resistance, including intrinsic resistance and acquired resistance after treatment with chemotherapeutic agents [182]. HMGA2 has been found to promote drug resistance by inherent intrinsic resistance and by inducing cancer stem cell populations. HMGA2 expression correlates with overall survival, as well as with progression-free survival (PFS) of patients who were treated with at least two cycles of cisplatin-based therapy [183]. Currently, it is understood that autophagy is critical in the process of cancer drug resistance in a variety of malignant cells [184]. In another study, a contradictory role was reported for HMGA2—Li et al. showed that HMGA2 overexpression suppresses gefitinib resistance in NSCLC cells by inhibiting autophagy [185]. They showed that HMGA2 overexpression decreased LC3B II expression, a key marker of autophagy, and subsequently reduced resistance to gefitinib by inhibiting autophagy.

It was identified that HMGA2 is a downstream target of let-7a to modulate cell proliferation, metastasis, and chemosensitivity to gemcitabine pancreatic cancer cells [186].

*miR-509-3* directly targets HMGA2 and *RAD51* enhances the sensitivity to Olaparib in ovarian cancer in both in vitro and in vivo experiments [187]. It is suggested that HMGA2, by regulating damage repair factors such as ATR, ATM, and ERCC, may induce resistance to Olaparib. Overexpression of HMGA2 enhances chemoresistance to 5-Fluorouracilfluorouracil (5-FU) via activation of the Wnt pathway in both in vitro and in vivo experiments of colorectal cancer. It was suggested that HMGA2 promotes Dvl2 expression, leading to enhanced activation of the Wnt/β-catenin pathway [188]. The inhibitory effect of the let-7g on the expression of the HMGA2 was demonstrated in a 5-FU-resistant human hepatoma cell line and it also reduced cyclin A expression, which is the key regulator of S phase and mitosis [189]. Additionally, our previous study showed that HMGA2 silencing using mature specific siRNAs sensitizes breast cancer cells to paclitaxel [190]. Lastly, in colorectal cancer cells, replacement of *miR-194* results in this miRNA targeting HMGA2 coexpressed genes through the TGFβ signaling pathway and this decreases cell viability in response to oxaliplatin and irinotecan [191]. These studies establish that HMGA2 plays an important role in cancer drug resistance by using different biological processes and this may act as a double-edged sword. HMGA2, by inhibiting autophagy, can decrease cancer drug resistance; however, most studies describe the significant role of HMGA2 in drug resistance by increasing cancer stem cell population, increasing DNA damage markers, and activating the Wnt-β catenin signaling pathway.

## 9. Perspectives and Conclusions

HMGA2 is a transcriptional regulator essential for embryonic development. Its expression is decreased during postembryonic development. However, HMGA2 levels are often re-expressed during oncogenesis. RNA sequencing analysis on clinical and normal samples has shown that HMGA2 is upregulated in most cancers. HMGA2 induces cancer cell proliferation by promoting entry into the S phase of the cell cycle. It also inhibits apoptosis and induces DNA repair. In addition, HMGA2 promotes EMT by inducing the MAPK/ERK, TGFβ/Smad and PI3K/AKT/mTOR/NFKB, and RKIP pathways, as well as regulating miRNA expression. HMGA2 also promotes cancer stem cell properties and induces drug resistance in cancer cells. Overall, HMGA2 may be used as a prognosis cancer marker, especially in aggressive stages of the disease. Additionally, HMGA2 may be a potential cancer therapeutic target. Targeting of HMGA2 via small interfering RNAs (siRNAs) or specific inhibitors including metformin could decrease cancer development, metastasis and drug resistance. In addition to the above-mentioned therapeutic strategies, miRNA replacement therapy has recently attracted significant attention and introducing the miRNAs which target the 3′UTR region of HMGA2 mRNA, such as those of the let-7 family, may be an attractive approach.

## Figures and Tables

**Table 1:** HMGA2 expression changes in different cancer types. Data compare cancer tissues to normal tissues. The cut-offs including *p*-value = 0.01, fold change = 1.5, and gene ranking = 10%.

Cancer	HMGA2 Status	Sample Size	Fold Change	*p*-Value	Reference
Bladder cancer	Down	60	−1.576	5.55 × 10^−8^	[37]
Brain and CNS cancer	Up	85	21.74	8.16 × 10^−5^	[38]
Breast cancer	Up	593	1.58	1.14 × 10^−4^	TCGA
	Up	66	1.57	0.003	[39]
	Down	59	−23.054	1.58 × 10^−23^	[40]
Colorectal cancer	Down	40	−2.51	1.15 × 10^−6^	[41]
Esophageal cancer	Up	34	5.093	5.32 × 10^−6^	[42]
	UP	106	1.785	1.06 × 10^−10^	[43]
Gastric cancer	Down	132	−1573	0.001	[44]
Head and neck cancer	UP	99	2.045	2.05 × 10^−6^	[45]
	UP	18	6.098	3.11 × 10^−5^	[46]
	UP	38	3.657	7.34 × 10^−7^	[47]
	UP	18	9.926	6.31 × 10^−6^	[48]
	UP	20	2.877	0.001	[49]
	UP	54	2.368	2.06 × 10^−7^	[50]
	UP	84	3.781	2.45 × 10^−5^	[51]
	UP	41	2.130	5.93 × 10^−5^	[52]
Leukemia	Down	293	−2.087	0.005	[53]
Lung cancer	Up	66	2.320	3.18 × 10^−5^	[54]
	Up	246	3.001	1.39 × 10^−12^	[55]
	Up	73	3.999	0.005	[56]
	Up	156	11.019	1.01 × 10^−10^	[57]
Lymphoma	Down	136	−1.798	1.14 × 10^−11^	[58]
Melanoma	Up	87	2.664	8.13 × 10^−4^	[59]
Ovarian cancer	Up	594	2.965	1.16 × 10^−5^	TCGA
Pancreatic cancer	Up	52	3.629	1.17 × 10^−7^	[60]
Prostate cancer	Up	15	126.524	1.15 × 10^−7^	[61]
	Down	35	−4.150	9.90 × 10^−6^	[62]
	Down	102	−1.681	0.007	[63]
Sarcoma	Up	158	2.565	3.55 × 10^−6^	[64]

TCGA: The Cancer Genome Atlas Program.

## Data Availability

Not applicable.

## Abbreviations

3′UTR: 3′-Three prime untranslated region, AKT: Protein Kinase B, ALDH: Aldehyde Dehydrogenase, AP: Apurinic/Apyrimidinic, AP1: Activator Protein-1, APE1: AP Endonuclease 1, Arg: Arginine, ATF/CREB: Activating Transcription Factor/cAMP Response Element (CRE)-Binding Protein, ATR: Ataxia Telangiectasia and Rad3-Related Kinase, BAD: BCL2-Associated Agonist of Cell Death, Bcl-2: B-Cell Lymphoma 2, BER: Base Excision Repair, BMI-1: B Lymphoma Mo-MLV Insertion Region 1, BRCA1: Breast Cancer Type 1, cAMP: Cyclic Adenosine Monophosphate, CCL5: C-C Motif Chemokine Ligand 5, CCNB2: Cyclin B2, CDK: Cyclin Dependent Kinase, CHK: Checkpoint Kinase 1, CRE: Cyclic AMP-Responsive Element, CREB: cAMP-Responsive Element Binding Protein, CXCR4: C-X-C Motif Chemokine Receptor 4, DNMT3A: DNA Methyltransferase 3 Alpha, E2F1: E2F Transcription Factor 1, EGF: Epidermal Growth Factor, EMT: Epithelial-Mesenchymal Transition, ERCC1: Excision Repair Cross-Complementation Group, ERK: Extracellular Receptor Kinase, ESC: Embryonic Stem Cell, FGF: Fibroblast Growth Factor, FOS: Fos Proto-Oncogene, FOXM1: Forkhead Box M1, FRA1: FOS-Like 1, GBM: Glioblastoma Multiforme, Gly: Glycine, HDAC1: Histone Deacetylase 1, HMG: High Mobility Group, HMGA2: High Mobility Group Protein 2, HNSCC: Head and Neck Squamous Cell Carcinoma, IGF2BP2: Insulin-Like Growth Factor 2 mRNA Binding Protein 2, IL-6: Interleukin 6, IMP2: Inner Mitochondrial Membrane Peptidase Subunit 2, Ink4a/Arf: Cyclin Dependent Kinase Inhibitor 2A, JUN: Jun Proto-Oncogene, Ku-80: ATP-Dependent DNA Helicase II 80 KDa Subunit, LC3B: Microtubule-Associated Protein 1 Light Chain 3 Beta, LOX: Lysyl Oxidase, MAPK: Mitogen-Activated Protein Kinase, MEK: Mitogen-Activated Protein Kinase, miRNA: MicroRNA, mTOR: Mechanistic Target of Rapamycin Kinase, MTSS1: MTSS I-BAR Domain Containing 1, NFkB: Nuclear Factor Kappa B, NHEJ: Nonhomologous End-Joining, NSCLC: Nonsmall-cell lung carcinoma, NSC: Neural Stem Cell, Oct4: Octamer-Binding Protein 4, p16INK4a: Cyclin Dependent Kinase Inhibitor 2A, P21CIP1: Cyclin Dependent Kinase Inhibitor 1A, PDGF-BB: Platelet-Derived Growth Factor-BB, PFS: Progression-Free Survival, PI3K: Phosphatidylinositol 3-kinase, PLAU: Plasminogen Activator, Urokinase, pRB: Retinoblastoma Protein, PRDII: Positive Regulatory Domain II, RAD51: DNA Repair Protein RAD51, RAF: Rapidly Accelerated Fibrosarcoma, RKIP: RAF Kinase Inhibitor Protein, SDC2: Syndecan-2, SIP-1: Smad-Interacting Protein-1, siRNA: Small Interfering RNA, SOX2: SRY-Box Transcription Factor 2, STAT3: Signal Transducer and Activator of Transcription 3, TGFβ: Transforming Growth Factor Beta, TNBC: Triple-Negative Breast Cancer, TNF: Tumor Necrosis Factor, TRAIL-R1: TNF-Related Apoptosis-Inducing Ligand-R2, VEGF: Vascular Endothelial Growth Factor, VEGFA: Vascular Endothelial Growth Factor A, WAF1: Wild-Type p53-Activated Fragment 1, ZEB1/2: Zinc Finger E-box Binding Protein 1/2, γ-H2AX: Phosphorylated form H2A Histone Family Member X, δEF-1: δ-Crystallin/E2-Box Factor 1.
